# What does the best interests principle of the convention on the rights of the child mean for paediatric healthcare?

**DOI:** 10.1007/s00431-022-04609-2

**Published:** 2022-09-09

**Authors:** Julian W. März

**Affiliations:** Institute of Biomedical Ethics and History of Medicine (IBME), University of Zurich, Winterthurerstrasse 30, 8006 Zurich, Switzerland

**Keywords:** Best interests principle, Convention on the Rights of the Child, Children’s rights, Paediatric ethics, Shared decision-making, Human rights in paediatrics

## Abstract

The present review analyses the implications of the best interests of the child principle, which is one of the most widely discussed principles of medical ethics and human rights, for paediatric healthcare. As a starting point, it presents the interpretation of the best interests principle by the United Nations Committee on the Rights of the Child. On this basis, it points out possible fields of application of the best interests principle with regard to paediatric healthcare and discusses the potential difficulties in the application of the best interests principle. Based on this, it illustrates the implications of the best interests principle for paediatric healthcare through four case studies, which look at ethical dilemmas in paediatric gynaecology, end-of-life care, HIV care and genetic testing.

*   Conclusion*: The best interests principle requires action, inter alia, by health policymakers, professional associations, hospital managers and medical teams to ensure children receive the best possible healthcare. Whilst the best interests principle does not provide a conclusive solution to all ethical dilemmas in paediatric healthcare (as illustrated by the case studies), it provides children, medical teams, parents and families, and clinical ethicists with an indispensable framework for health care centred on the rights of the child.

**Table:** 

**What is Known:** *• The best interests principle is one of the most widely discussed principles of medical ethics and human rights and one of the four general principles of the Convention on the Rights of the Child.*	
**What is New:** *• The present review discusses possible fields of application and potential difficulties of the best interests principle with regard to paediatric healthcare.* •* Based on this, it illustrates the implications of the best interests principle for paediatric healthcare through four case studies, which look at ethical dilemmas in paediatric gynaecology, end-of-life care, HIV care and genetic testing.*

**What is Known:**

*• The best interests principle is one of the most widely discussed principles of medical ethics and human rights and one of the four general principles of the Convention on the Rights of the Child.*

**What is New:**

*• The present review discusses possible fields of application and potential difficulties of the best interests principle with regard to paediatric healthcare.*

•* Based on this, it illustrates the implications of the best interests principle for paediatric healthcare through four case studies, which look at ethical dilemmas in paediatric gynaecology, end-of-life care, HIV care and genetic testing.*

## Introduction

The best interests of the child principle is one of the most widely discussed principles of medical ethics and human rights (Table 1). It is one of the four general principles of the Convention on the Rights of the Child^1^ (Table 2), which states that “in all actions concerning children […], the best interests of the child shall be a primary consideration”.

**Table 1:** Selected examples of medical ethics and human rights articles discussing implications of the best interests principle for paediatric healthcare

Public health interventions
• Compulsory vaccination of children [47–51]
• Provision of needle and syringe services for adolescents [52]
• Newborn screening [53, 54]
• Rationing of medical resources [55, 56]
Sexual and reproductive health
• Reproductive and sexual health education [57]
• Paediatric HIV/AIDS disclosure [33]
• Ovarian or testicular tissue cryopreservation [58–60]
• Access to gender-affirming or puberty-blocking medication for transgender and intersex adolescents [61, 62]
• Posthumous medically assisted reproduction [63]
• Gamete donation [64]
Genetic testing
• Clinical genomics [65, 65–75]
• Preimplantation genetic testing [76]
• Saviour siblings [77–79]
Decisions to provide or withhold medical treatment
• Paediatric intensive care [80]
• Blood transfusion to Jehovah’s Witness children [81, 82]
• Withholding or withdrawing medical treatment [83–85, 83–93]
• Treatment of extremely premature infants [94, 55]
• Conjoint twins surgery [95]
• Elective paediatric surgery [96]
Clinical research and experimental interventions
• Participation of children in clinical research [97–107]
• Experimental treatment options [108–111]
• Off-label use of medicines [112]
• Cognitive enhancement [113, 114]
• Genetic enhancement [115]
Interaction of healthcare professionals with children
• Development of participatory approaches to paediatric healthcare [116, 117]
• Development of paediatric cancer nursing interventions [118]
• Treatment of psychiatrically ill children [119]
• Determination of capacity to consent to medical treatment [120]
• Interventions to address child maltreatment [121, 122]
Other topics
• Intersex paediatric surgery [123–128]
• Bone marrow donation (to a sibling) [129–132]
• Medical tourism [133]
• Home birth [134]
• Complementary therapies [135, 136]

**Table 2:** General principles of the Convention on the Rights of the Child (CRC) [1, 137]

Non-discrimination
Article 2 CRC: “States Parties shall respect and ensure the rights set forth in the present Convention to each child within their jurisdiction without discrimination of any kind, irrespective of the child’s or his or her parent's or legal guardian’s race, colour, sex, language, religion, political or other opinion, national, ethnic or social origin, property, disability, birth or other status.”
Best interests principle
Article 3 CRC: “In all actions concerning children, whether undertaken by public or private social welfare institutions, courts of law, administrative authorities or legislative bodies, the best interests of the child shall be a primary consideration.”
Right to life, survival and development
Article 6 CRC: “States Parties recognize that every child has the inherent right to life. States Parties shall ensure to the maximum extent possible the survival and development of the child.”
Right to freely express his or her views and to have them heard
Article 12 CRC: “States Parties shall assure to the child who is capable of forming his or her own views the right to express those views freely in all matters affecting the child, the views of the child being given due weight in accordance with the age and maturity of the child.”

The present review analyses the implications of the best interests principle for paediatric healthcare. As a starting point, it presents the interpretation of the best interests principle by the United Nations Committee on the Rights of the Child.^2^ On this basis, it points out possible fields of application of the best interests principle with regard to paediatric^3^ healthcare and discusses the potential difficulties in the application of the best interests principle. Based on this, it illustrates the implications of the best interests principle for paediatric healthcare through four case studies, which look at ethical dilemmas in paediatric gynaecology, end-of-life care, HIV care and genetic testing.

## Interpretation of the best interests principle by the United Nations committee on the rights of the child

The United Nations Committee on the Rights of the Child has been created by the Convention on the Rights of the Child as the main institution to monitor its implementation. Through its currently 25 general comments, the committee provides authoritative guidance to the state parties on the implementation of the Convention on the Rights of the Child. In addition, it provides individual guidance to specific countries through its concluding observations on the periodic reports which all state parties to the Convention on the Rights of the Child have to submit every 5 years.

According to the Committee on the Rights of the Child, the best interests principle has a threefold function as a substantive right, a fundamental legal principle and a rule of procedure [5, 6]. Table 3 explains this threefold function using examples from the context of paediatric healthcare.

**Table 3:** Different dimensions of the best interests principle (with examples) [6]

Substantive right: Children (both as individuals and as a group) have a right to have their bests interests taken into account as the primary consideration in all decisions affecting them.
• Medical decisions affecting a child must be preceded by a best interests assessment.
• Children should not be separated from their parents or primary caregivers (e.g. in a context of paediatric hospitalization) unless for imperative and inevitable reasons.
• Adequate emotional care for children must be ensured, notably in cases of children suffering from life-threatening diseases (e.g. cancer).
• Paediatric healthcare services should receive sufficient funding to offer high-quality medical services.
Interpretative legal principle: All laws and guidelines (e.g. paediatric treatment guidelines) must be interpreted and applied in line with the best interests principle.
• Paediatric treatment decisions should not be (primarily) based on economic considerations.
• Hospital policies (e.g. visit policies) should be applied in line with the best interests principle.
Procedural rule: Best interests assessments should be integral parts of important decision-making processes in paediatric healthcare.
• Children should be asked for their views on treatment decisions, which should be adequately considered and taken seriously.
• The child and his or her parents and family should be informed and involved into all steps of the decision-making process (shared decision-making).
• Medical teams should receive adequate training in paediatric ethics and children’s rights.
• Healthcare service providers (e.g. hospitals) should have strict preventive policies against child abuse, and should adequately train their staff on this topic.
• All policy measures affecting paediatric healthcare should be subject to a child-rights impact assessment (CRIA).

The obligation to give primary consideration to a child’s best interests applies to all private and public organizations whose decisions can potentially impact children [6]. This means not only that all policies relating to the child (including health policy) have to abide by the best interests principle, but also that all (paediatric) healthcare providers have to give due consideration to children’s best interests in all their decisions (potentially) affecting children, which is illustrated by examples in Table 4.

**Table 4:** Examples of best interests obligations on different levels (in relation to paediatric healthcare) (based on [6, 7, 10, 25, 34, 35, 138–145])

All levels
• Development and implementation of policies to combat violence against children (including female genital mutilation and intersex gender assignment surgery for non-medical reasons) and child abuse
• Development and implementation of policies to address infectious diseases (notably HIV/AIDS) in children
• Awareness-raising and (regulatory) measures to address unhealthy lifestyles in children (e.g. alcohol, drug and tobacco use; overweight and lack of physical exercise)
• Measures to ensure equal access of marginalized groups of children (e.g. children with disabilities; children living with HIV/AIDS; children in street situations; unaccompanied and separated migrant children) to healthcare services and to address their specific healthcare needs
• Development and implementation of policies to address child bullying and discrimination
Health policy
• Development of age-appropriate information campaigns on sexual and reproductive health
• Development of child mental health services, particularly for survivors of violence and abuse
• Adequate funding of paediatric healthcare
• Investment in digital health services and digital skills for children
• Collection of data and development of research programmes on child health, particularly with regard to marginalized groups of children
Professional organizations (e.g. paediatricians’ or nurses’ associations)
• Development of awareness and training programmes on violence against children and child abuse
• Development of training programmes on healthcare for marginalized groups of children
• Development of clinical guidelines on the implementation of the best interests principle in practice
• Development of education programmes on children’s rights in healthcare
Hospital management
• Sensibilization of staff on child abuse, violence against children and discriminatory practices
• Development of hospital policies in line with the best interests principle
• Creation of a clinical ethics board to assist in ethically difficult cases (e.g. end-of-life paediatric care)
Medical team
• Participation in regular training programmes on violence against children, child abuse and healthcare for marginalized groups of children
• Non-discriminatory provision of healthcare services
• Respect for the best interests principle as a major guideline for paediatric healthcare

## Application of the best interests principle in paediatric healthcare

Table 1 summarizes possible areas of application of the best interests principle in paediatric healthcare discussed in the children’s rights and paediatric ethics literature.

### Importance to respect the child’s views

The child’s right to have his or her views duly taken into account is one of the four general principles of the Convention on the Rights of the Child (Table 1). Even though children (at least up until adolescent age) are as a general rule incapable (in a legal sense) to make important medical decisions themselves, this does not mean that their perspectives and views are irrelevant. On the contrary, as the United Nations Committee on the Rights of the Child emphasizes, “[t]he realization of the provisions of the Convention requires respect for the child’s right to express his or her views and to participate in promoting the healthy development and well-being of children. This applies to individual health-care decisions, as well as to children’s involvement in the development of health policy and services” [7]. Particularly in the case of older children, medical decisions should not be made about them, but rather in partnership with them. But even in the case of younger children, medical professionals should try to involve them as much as possible in the decision-making process, at the very least by explaining which treatments are carried out and for which reason and by trying to obtain their assent for a medical intervention. Even though this might be a burdensome task in some cases and have no effect on the immediate treatment decision, it is an important step in the child’s development towards a person capable to take his or her own decisions about his or her health.

### Capacity to consent to medical interventions

A particularly problematic legal issue is the question when a child has achieved a sufficient level of competency and maturity to take certain health-related decisions independently for himself or herself. The United Nations Committee on the Rights of the Child recommends that states “review or introduce legislation recognizing the right of adolescents to take increasing responsibility for decisions affecting their lives […] [and] introduce minimum legal age limits,^4^ consistent with the right to protection, the best interests principle and respect for the evolving capacities of adolescents, […] [which] should recognize the right to make decisions in respect of health services or treatment”; furthermore, “the right of any child below that minimum age and able to demonstrate sufficient understanding to be entitled to give or refuse consent should be recognized” [10]. In addition, the United Nations Committee on the Rights of the Child states that adolescents have a right to access sexual and reproductive health services and to obtain confidential medical counselling without a parent’s consent [10].^5^ Moreover, it recommends a decriminalization of abortion and a review of abortion-related legislation under the best interests principle, which should ensure that the pregnant adolescent’s views are always respected in decisions related to abortion [10].

### Case study 1: Access of a 14-year-old girl to contraceptive treatment^6^

“A”, a 14-year-old girl, is visiting her gynaecologist since she is suffering from menorrhagia (heavy menstrual bleeding), which causes mild anaemia and painful menstrual cramps. Given her young age and the potential side effects of oral contraceptive treatment, the treating physician recommends the implantation of a levonorgestrel-releasing intrauterine device (IUD). “A” refuses the implantation of an IUD due to fear of injuries and prefers oral contraception to treat her menorrhagia. Her parents consider her too young for contraceptive treatment and only wish for symptomatic treatment for the moment.

Access to sexual and reproductive health services is a fundamental human right, which is recognized, for instance, by the United Nations Committee on the Rights of the Child [10], the United Nations Committee on Economic, Social and Cultural Rights [11, 12] and the United Nations Committee on the Elimination of Discrimination against Women [13]. The United Nations Committee on the Rights of the Child has repeatedly condemned states’ efforts to bar adolescents from access to contraceptive advice and treatment (e.g. [14]). In the present case, problems arise on the one hand due to a disagreement between the treating physician and the patient on the preferable form of contraceptive treatment, on the other hand, due to a profound disagreement of the parents with contraceptive treatment for their child in general. In this situation, acting in the child’s best interests implies respecting her views and experiences, giving due consideration to her need for information and treatment wishes and tailoring medical counselling accordingly. The physician should also try to involve A’s parents as much as possible and explain to them the rationale for using contraceptive treatment in this case. Finally, the best interests principle also applies at the institutional level, meaning that physicians, particularly gynaecologists and paediatricians, should receive adequate training and be able to access information (e.g. clinical guidelines) on adolescents’ sexual and reproductive health. On the issue of contraception, various national paediatrics and gynaecology organizations have published guidance, e.g. [15–17].

### Shared responsibility for ensuring children’s best interests

Article 7 of the Convention on the Rights of the Child guarantees the child’s right “to be cared for by his or her parents”. As Pruski and Gamble point out, responsible and caring parents should in general be presumed to make decisions which are in the best interests of their children [18]. There are, however, situations in which decision-making for a child can be a heavy burden, both due to emotional challenges and medical complexity. For instance, end-of-life decisions are a particular challenge for the child concerned, his or her parents and family and also the treating medical staff. Whilst consensus can often be reached between the parents, the families and the treating physicians, this is not always the case, as in the case study discussed below. In these situations, the medical team, the parents and the family should be aware of the fact that a supporting family environment and a stable and trusted doctor-patient-relationship are in general required to offer the child the best possible treatment [19].

### Who should decide what is in a child’s best interests?

A particularly complex ethical and legal problem is the issue who should (and could) decide what is in a child’s best interests. Whilst consensus between the child and all other stakeholders (including the family and the medical team) is in general desirable, it is sometimes not possible to establish. For the medical team, it can be challenging to decide when the time has come to give up efforts to reach a consensus with the parents (and the child) and to refer a matter to the courts for resolution. Whilst court proceedings can seriously undermine the relationship between the child, the parents and the medical team, they also offer the possibility of an objective and impartial review of the case. In addition, court proceedings can relieve the parents and the medical team from the burden of decision-making for the child in a situation of extreme distress. The United Nations Committee on the Rights of the Child thus considers that the best interests principle implies that “[s]tates must put in place formal processes, with strict procedural safeguards, designed to assess and determine the child’s best interests for decisions affecting the child, including mechanisms for evaluating the results” [6]. Court procedures should, in particular, leave sufficient room for the child to express his or her own views, include an establishment of the facts and an assessment of the consequences of the decision taken by experts and include the possibility for a review of the court’s best interests assessment (e.g. through an appeal or the need for a reapplication if the factual circumstances change) [6].

### Case study 2: Treatment refusal by the parents of a 6-year-old child

“B”,^7^ a 6-year-old boy, suffered from medulloblastoma, a rare brain tumour. Upon the recommendation of his physicians and with the consent of his parents, he had undergone tumour surgery, which had not cured his disease. Therefore, his physicians recommended chemoradiation, which was estimated to give “B” a chance of 5-year survival of between 30 and 60%. The parents, however, refused this treatment since they feared the suffering of their child from the side effects of chemoradiation and thus preferred palliative treatment.^8^

Decisions about the withholding or withdrawing of paediatric treatment count amongst the ethically and emotionally most difficult decisions in healthcare.^9^ Estimates for the UK suggest that 49,000 children are suffering from conditions which might necessitate end-of-life decisions [26]. Furthermore, it is estimated that around 80% of deaths in paediatric intensive care are linked to (yet not caused by) decisions to withhold or withdraw medical treatment [27].

Situations like in B’s case are extremely distressing for all persons involved—the child, his or her parents and family and the medical team. Deciding what best to do in a case such as this is certainly not an easy task. A discussion of the treatment options and the medical background between the medical team, the child, his or her parents and other family and the clinical ethics unit should be the first step. For the medical team, it is essential to know and to respect the views, values and evaluation of the parents and the child, and vice versa. Successful medical treatment of a child is often impossible without a strong and supporting family [19] and a consensus between the medical staff, the child and his or her parents and family. Shared-decision making, which integrates the parents and the child into the decision-making process, can also help parents to cope better with the burden of having to make the best decision for their child [28].

### Need for a biopsychosocial assessment of a child’s best interests

Clinical guidelines provide guidance for evidence-based medical treatment. High standards in paediatric healthcare are, of course, an important requirement of the best interests principle. However, providing the best available medical treatment is not always tantamount to respect for a child’s best interests. According to the United Nations Committee on the Rights of the Child, a best interests assessment needs to consider the situation of a child as an individual [6]. This means taking into account not only medical, but also psychological and social factors. This has, for instance, been recognized in the context of bone marrow donation by a child to a sibling: Although the bone marrow donation does not yield a direct medical advantage to the child donor, it can nevertheless be in his or her best interests if it is necessary to avoid the death of the sibling and the suffering of the family [29].

### Case study 3: Paediatric HIV disclosure

“C”, a 3-year-old boy, has recently emigrated with his mother from Sierra Leone to the UK, where both have been diagnosed with HIV. Both are currently under combination antiretroviral therapy (cART), under which their CD4 count has stabilized. The issue now arises when and in which form his HIV infection should be communicated to “C”.

According to UNAIDS estimates, around 1.7 million children worldwide below the age of 15 are living with HIV [30]. In the UK, around 300 children below the age of 15 are currently receiving cART [31]. Whilst there are effective treatments to prevent mother-to-child HIV transmission, mother-to-child HIV transmission continues to occur, particularly in cases where the mother’s HIV infection had not yet been diagnosed during pregnancy.

Disclosure of a life-threatening and life-changing disease to a child poses significant ethical problems. Even if treatment options for paediatric HIV infections are available, mortality rates are still 30-fold if compared to the general population [32]. Children living with HIV also face significant risks of stigmatization and social exclusion.

Studies have generally shown that children benefit from early HIV disclosure [33]. Information should be tailored to a child’s maturity and understanding and should respect the child’s wishes as to the amount and kind of information provided as much as possible. Parents or primary caregivers should also be involved in the discussions as much as possible. At the institutional level, acting in the child’s best interests means ensuring the confidentiality of the diagnosis, and passing it on to other persons (e.g. other healthcare professionals) only with the child’s or his or her parents’ consent or for imperative public health reasons. Finally, as the United Nations Committee on the Rights of the Child underlines, states are under an obligation to provide “legal, economic and social protection to affected children to ensure their access to education, inheritance, shelter and health and social services, as well as to make them feel secure in disclosing their HIV status and that of their family members when the children deem it appropriate” [34].

### The duty to protect a child’s autonomy

According to the Convention on the Rights of the Child, one of the key rationales of children’s rights is to ensure “that the child should be fully prepared to live an individual life in society, and brought up in the spirit of the ideals proclaimed in the Charter of the United Nations, and in particular in the spirit of peace, dignity, tolerance, freedom, equality and solidarity” [1]. Childhood is part of a development process, at the end of which the child should have become a competent adult, who is capable of taking autonomous decisions about his or her health. As set out by Article 5 of the Convention on the Rights of the Child, decisions regarding the child should give due consideration to the child’s “evolving capacities”, which, according to the United Nations Committee on the Rights of the Child, “should be seen as a positive and enabling process, not an excuse for authoritarian practices that restrict children’s autonomy and self-expression and which have traditionally been justified by pointing to children’s relative immaturity and their need for socialization” [35]. This means that decisions which preclude a child’s future autonomy to decide freely about his or her health and body are not in the best interests of that child. For instance, predictive testing for adult-onset genetic diseases, e.g. hereditary breast and ovarian cancer (HBOC), is in general not in the best interests of a child [36, 37]. Gender assignment surgery in intersex children (unless for urgent medical reasons), which has been strongly condemned by the United Nations Committee on the Rights of the Child (e.g. [10, 38–43]) and many other United Nations and regional human rights treaty bodies [44], is also a clear violation of the best interests principle.

### Case study 4: Genetic testing of an adolescent for a genetic cancer predisposition

“D” is a 15-year-old girl who seeks medical advice due to a family history of breast cancer. Her mother died of breast cancer at age 36 3 months beforehand. A half-sister and an aunt have also died of breast cancer at young ages. In all three relatives, *BRCA2* mutations have been detected, which increase the lifetime risk of breast cancer to around 60–70% and of ovarian cancer to 20–30%. “D” has engaged extensively with (lay) literature on hereditary breast cancer and *BRCA2* and asks for genetic testing for the known familial *BRCA2* mutation.

Predictive genetic testing in children carries significant ethical challenges. The results of genetic tests can cause significant emotional distress and impact life choices. Since the child will be the person who will have to cope with the test results, it is generally advised that the decision for predictive genetic testing should be taken by the child once he or she is competent to take this decision, unless the test should be taken at an earlier age to avoid serious harm to the child’s health. Therefore, clinical guidelines in general discourage from testing for *BRCA2* mutations in persons below age 18, given that screening for breast cancer in *BRCA2* carriers (e.g. MRI scans of the breast) is generally only offered at age 25 and older [45, 46]. This should be part of a detailed discussion of the social, medical and psychological implications of testing for *BRCA2* carrier status with “D”. If the physician decides to proceed with genetic testing,^10^ he or she should ensure that “D” disposes of sufficient knowledge and the intellectual capabilities to understand what the procedure and results of a genetic test for hereditary breast and ovarian cancer involve. He or she should make sure that “D” receives sufficient information to interpret and process the results of the genetic test and can access psychosocial counselling should she require it.

## Conclusion

With the words of the United Nations Committee on the Rights of the Child, the best interests principle is “one of the fundamental values of the Convention [on the Rights of the Child] […] [which] requires the development of a rights-based approach, engaging all actors, to secure the holistic physical, psychological, moral and spiritual integrity of the child and promote his or her human dignity” [6]. It requires action, inter alia, by health policymakers, professional associations, hospital managers and medical teams to ensure children receive the best possible healthcare. Acting in the best interests of the child means respecting the child as a person who, with the words of the Convention on the Rights of the Child, “deserves to be raised in the spirit of peace, dignity, tolerance, freedom, equality and solidarity” [1]. Whilst the best interests principle does not provide a conclusive solution to all ethical dilemmas in paediatric healthcare (as illustrated by the case studies), it provides children, medical teams, parents and families, and clinical ethicists with an indispensable framework for health care centred on the rights of the child.

## Footnotes

1 The Convention on the Rights of the Child of 20 November 1989 [1] is the most widely accepted international human rights treaty. As of March 2022, it has been ratified by all member states of the United Nations, with the sole exception of the United States [2]. For a more detailed presentation of children’s rights in paediatric healthcare, see [3].

2 The present article discusses primarily the interpretation of the best interests principle by the United Nations Committee on the Rights of the Child, with references to national law where appropriate. Note that national legal rules on best interests of the child issues, e.g. capacity to consent to medical treatments, paediatric treatment withdrawal or conditions for elective paediatric surgery, vary widely between countries.

3 The present review uses the understanding of “paediatrics” of the American Academy of Pediatrics, which sets the upper age limit at 21 years, allowing exceptions if the family and the paediatrician agree, in particular in cases of children with special health needs [4].

4 In England and Wales, for instance, an adolescent aged above 16 is deemed competent to consent to medical treatment (s. 8 Family Law Reform Act 1969). A child below the age of 16 can be deemed legally competent to consent to medical treatment if he or she is “capable of understanding what is proposed, and of expressing his or her own wishes, […] validly and effectively” (*Gillick* case [8]). It is primarily a task of the treating physician to assess if a child has sufficient understanding and intellectual maturity to understand a medical treatment, particularly its risks (*Bell* v *Tavistock* [9]). Similarly, s. 2, para. 4 Age of Legal Capacity (Scotland) Act 1991 provides that a child under the age of 16 has legal capacity to consent to medical treatment if, “in the opinion of a qualified medical practitioner attending him, he is capable of understanding the nature and possible consequences of the procedure or treatment”.

5 The English courts are significantly more restrictive with regard to access to sexual and reproductive health services and information by adolescents than the United Nations Committee on the Rights of the Child. In the *Gillick* case [8], the House of Lords has set out five conditions which must be fulfilled before physicians can provide sexual and reproductive health advice to adolescents: First, the physician must satisfy himself or herself that the adolescent will understand the advice provided. Second, the physician must try to persuade the adolescent to inform his or her parents that he or she is seeking sexual and reproductive advice. Third, the physician must reach the conclusion that the adolescent would start or continue having sexual intercourse even without contraception. Fourth and fifth, the physician concludes that the adolescent’s physical or mental health would be harmed without access to contraceptive advice or treatment and that receiving this advice or treatment without the parents’ consent is in the best interests of the child (*Gillick* [8], at p. 413).

6 I am grateful to the anonymous peer reviewer who suggested the inclusion of this case study into the article.

7 The case study is based on the *Child and Adolescent Health Services (CAHS)* v *Kiszko & Anor* case [20–22]. A detailed presentation and discussion of the case is provided by [23, 24].

8 Note that the “standard” situation is often reversed, meaning that the parents ask for additional treatment of their child whilst the medical team sees no point in continuing medical treatment. A number of highly mediatized cases of treatment withdrawal against the wishes of the parents have been litigated in recent years, e.g. the cases of Charlie Gard (2017), Alfie Evans (2018) and Archie Battersbee (2022) in the UK.

9 One of the reviewers has raised the point that rationing pressures, which exist in many healthcare systems, mean that the interests of one child to receive a particularly costly treatment must also be weighed against the interests of children in general. Whilst this contention is correct, it should also be noted that the best interests principle requires states to allocate sufficient funds to paediatric healthcare services in order to avoid rationing pressures as much as possible and to allow, as much as feasible, treatment options to be taken independently from economic considerations [25].

10 Note, however, that genetic testing for hereditary breast and ovarian cancer is illegal in persons below age 18 in some jurisdictions, e.g. Germany.

## References

[1] Convention on the Rights of the Child of 20 November 1989. UN General Assembly Resolution 44/25

[2] Office of the United Nations High Commissioner for Human Rights Ratification Status for CRC - Convention on the Rights of the Child. Available at: https://tbinternet.ohchr.org/_layouts/15/TreatyBodyExternal/Treaty.aspx?Treaty=CRC&Lang=en. Accessed 7 Apr 2022

[3] Streuli JC, Michel M, Vayena E (2011). Children’s rights in pediatrics. Eur J Pediatr.

[4] Hardin AP, Hackell JM (2017) Age limit of pediatrics. Pediatrics 140(3). 10.1542/peds.2017-2151 10.1542/peds.2017-2151 28827380

[5] Kilkelly U (2016) The best interests of the child: a gateway to children’s rights? In: Sutherland EE, Macfarlane L-AB (eds) Implementing Article 3 of the United Nations Convention on the Rights of the Child. Cambridge University Press, Cambridge, pp 51–66

[6] United Nations Committee on the Rights of the Child (2013) General comment No. 14 on the right of the child to have his or her best interests taken as a primary consideration (CRC/C/GC/14)

[7] United Nations Committee on the Rights of the Child (2009) General comment No. 12: the right of the child to be heard (CRC/C/GC/12)

[8] House of Lords (1985) Gillick v West Norfolk and Wisbech Area Health Authority and another [1985] 3 All ER 402

[9] Court of Appeal of England and Wales (2021) Bell v Tavistock [2021] EWCA Civ 1363

[10] United Nations Committee on the Rights of the Child (2016) General comment No. 20 on the implementation of the rights of the child during adolescence (CRC/C/GC/20)

[11] United Nations Committee on Economic, Social and Cultural Rights (2000) General comment No. 14 on the right to the highest attainable standard of health (article 12 of the International Covenant on Economic, Social and Cultural Rights) (E/C.12/2000/4)

[12] United Nations Committee on Economic, Social and Cultural Rights (2016) General comment No. 22 on the right to sexual and reproductive health (article 12 of the International Covenant on Economic, Social and Cultural Rights) (E/C.12/GC/22)

[13] United Nations Committee on the Elimination of Discrimination against Women (1999) General recommendation No. 24 on women and health (U.N. Doc. A/54/38)

[14] United Nations Committee on the Rights of the Child (2021) Concluding observations on the combined fifth and sixth periodic reports of Poland (CRC/C/POL/CO/5–6)

[15] American Academy of Pediatrics - Committee on Adolescence (2014). Contraception for adolescents. Pediatrics.

[16] American College of Obstetricians and Gynecologists (2017) Counseling adolescents about contraception. Available at: https://www.acog.org/clinical/clinical-guidance/committee-opinion/articles/2017/08/counseling-adolescents-about-contraception. Accessed 23 August 2022

[17] Faculty of Sexual & Reproductive Healthcare (2019) Clinical guidance - contraceptive choices for young people. Available at: https://www.fsrh.org/standards-and-guidance/fsrh-guidelines-and-statements/contraception-for-specific-populations/young-people/. Accessed 23 August 2022

[18] Pruski M, Gamble NK (2019). Reasonable parental and medical obligations in pediatric extraordinary therapy. Linacre Q.

[19] Kearney PJ (1978). Leukaemia in children of Jehovah’s Witnesses: issues and priorities in a conflict of care. J Med Ethics.

[20] Child and Adolescent Health Services (CAHS) v Kiszko & Anor (No. 2) (2016) FCWA 34

[21] Child and Adolescent Health Services (CAHS) v Kiszko & Anor (No. 1) (2016) FCWA 19

[22] Child and Adolescent Health Services (CAHS) v Kiszko & Anor (No. 3) (2016) FCWA 75

[23] Okninski M (2016). Determining a child’s best interests when parents refuse medical treatment-CAHS v Kiszko & Anor 2016 FCWA 19. Journal of bioethical inquiry.

[24] Richards BJ, Okninski ME (2017). The best interests of a child: a tragedy in three parts: (CAHS V Kiszko & Anor 2016 FCWA 19, CAHS v Kiszko & Anor 2016 FCWA 34 and CAHS v Kiszko & Anor 2016 FCWA 75). Med Law Rev.

[25] United Nations Committee on the Rights of the Child (2013) General comment No. 15 on the right of the child to the enjoyment of the highest attainable standard of health (CRC/C/GC/15)

[26] Moreton KL (2020). Reflecting on ‘Hannah’s choice’: using the ethics of care to justify child participation in end of life decision-making. Med Law Rev.

[27] Inwald D (2008). The best interests test at the end of life on PICU: a plea for a family centred approach. Arch Dis Child.

[28] Sullivan J, Gillam L, Monagle P (2015). Parents and end-of-life decision-making for their child: roles and responsibilities. BMJ Support Palliat Care.

[29] Re Y (Mental patient: bone marrow donation) (1997) Fam 110

[30] UNAIDS Fact Sheet (2022) Global HIV Statistics. Available at: https://www.unaids.org/en/resources/fact-sheet. Accessed 23 August 2022

[31] National AIDS Trust (2019) HIV in the UK statistics. Available at: https://www.nat.org.uk/about-hiv/hiv-statistics. Accessed 23 August 2022

[32] Davies M-A, Gibb D, Turkova A (2016). Survival of HIV-1 vertically infected children. Curr Opin HIV AIDS.

[33] Lesch A, Swartz L, Kagee A, Moodley K, Kafaar Z, Myer L, Cotton M (2007). Paediatric HIV/AIDS disclosure: towards a developmental and process-oriented approach. AIDS Care.

[34] United Nations Committee on the Rights of the Child (2003) General comment No. 3 on HIV/AIDS and the rights of the child (CRC/GC/2003/3)

[35] United Nations Committee on the Rights of the Child (2005) General comment No. 7: implementing child rights in early childhood (CRC/C/GC/7/Rev.1)

[36] Committee on Bioethics, Committee on Genetics, and the American College of Medical Genetics and Genomics Social, Ethical, and Legal Issues Committee,  (2013). Ethical and policy issues in genetic testing and screening of children. Pediatrics.

[37] Ross LF, Saal HM, David KL, Anderson RR (2013). Technical report: ethical and policy issues in genetic testing and screening of children. Genet Med.

[38] United Nations Committee on the Rights of the Child (2016) Concluding observations on the fifth periodic report of the United Kingdom of Great Britain and Northern Ireland (CRC/C/GBR/CO/5)

[39] United Nations Committee on the Rights of the Child (2019) Concluding observations on the combined fifth and sixth periodic reports of Australia (CRC/C/AUS/CO/5–6)

[40] United Nations Committee on the Rights of the Child (2019) Concluding observations on the combined fifth and sixth periodic reports of Portugal (CRC/C/PRT/CO/5–6)

[41] United Nations Committee on the Rights of the Child (2020) Concluding observations on the combined fifth and sixth periodic reports of Austria (CRC/C/AUT/CO/5–6)

[42] United Nations Committee on the Rights of the Child (2021) Concluding observations on the combined fifth and sixth periodic reports of Switzerland (CRC/C/CHE/CO/5–6)

[43] United Nations Committee on the Rights of the Child (2022) Concluding observations on the combined fifth and sixth periodic reports of the Kingdom of the Netherland (CRC/C/NLD/CO/5–6)

[44] Office of the United Nations High Commissioner for Human Rights (2016) End violence and harmful medical practices on intersex children and adults, UN and regional experts urge. Available at: https://www.ohchr.org/en/2016/10/intersex-awareness-day-wednesday-26-october?LangID=E&NewsID=20739. Accessed 7 Apr 2022

[45] Elger BS, Harding TW (2000). Testing adolescents for a hereditary breast cancer gene (BRCA1) - respecting their autonomy is in their best interest. Arch Pediatr Adolesc Med.

[46] NIH National Cancer Institute (2020) BRCA gene mutations: cancer risk and genetic testing. Available at: https://www.cancer.gov/about-cancer/causes-prevention/genetics/brca-fact-sheet. Accessed 23 August 2022

[47] Archard D, Brierley J, Cave E (2021). Compulsory childhood vaccination: human rights, solidarity, and best interests. Med Law Rev.

[48] Bayefsky MJ (2018). The ethical case for mandating HPV vaccination. Journal of law, medicine & ethics.

[49] Bester JC (2017). Measles vaccination is best for children: the argument for relying on herd immunity fails. Journal of bioethical inquiry.

[50] Hodges FM, Svoboda JS, van Howe RS (2002). Prophylactic interventions on children: balancing human rights with public health. J Med Ethics.

[51] Pierik R (2020). Vaccination policies: between best and basic interests of the child, between precaution and proportionality. Public health ethics.

[52] Barrett D, Petersson F, Turner R (2022). Best interests and low thresholds: legal and ethical issues relating to needle and syringe services for under 18s in Sweden. Harm Reduct J.

[53] Farrell PM (2008). Is newborn screening for cystic fibrosis a basic human right?. Journal of cystic fibrosis: official journal of the European Cystic Fibrosis Society.

[54] Knoppers BM, Sénécal K, Borry P, Avard D (2014) Whole-genome sequencing in newborn screening programs. Science translational medicine 6(229):229cm2. 10.1126/scitranslmed.3008494 10.1126/scitranslmed.3008494 24670681

[55] Haward MF, Janvier A, Moore GP, Laventhal N, Fry JT, Lantos J (2020). Should extremely premature babies get ventilators during the COVID-19 crisis?. Am J Bioeth.

[56] Kirby L, Basu S, Close E, Jansen M (2021). Rationing in the pediatric intensive care unit-ethical or unethical?. Translational pediatrics.

[57] Bridgeman J (2006) Young people and sexual health: whose rights? Whose responsibilities? R. (on the application of Axon) v. Secretary of State for Health & another. Med Law Rev 14(3):418–424. 10.1093/medlaw/fwl014 10.1093/medlaw/fwl014 16963473

[58] Affdal AO, Ravitsky V (2018). The best interest standard and the child’s right to an open future. Am J Bioeth.

[59] Di Pietro ML, Teleman AA (2013). Cryopreservation of testicular tissue in pediatrics: practical and ethical issues. J Matern Fetal Neonatal Med.

[60] Di Pietro ML, Virdis A, Gonzalez-Melado FJ, de Luca D (2012). Cryopreservation of ovarian tissue in pediatrics: what is the child’s best interest?. J Matern Fetal Neonatal Med.

[61] Beattie C (2022). High court should not restrict access to puberty blockers for minors. J Med Ethics.

[62] Kon AA (2014). Transgender children and adolescents. Am J Bioeth.

[63] Pobjoy J (2007). Medically mediated reproduction: posthumous conception and the best interests of the child. J Law Med.

[64] Takes F (2022). The child’s best interest in gamete donation. Bioethics.

[65] Bush L (2014). In the best interest of the child: psychological and ethical reflections on traditions, contexts, and perspectives in pediatric clinical genomics. Am J Bioeth.

[66] Camporesi S, McNamee MJ (2016). Ethics, genetic testing, and athletic talent: children’s best interests, and the right to an open (athletic) future. Physiol Genomics.

[67] Clayton EW, McCullough LB, Biesecker LG, Joffe S, Ross LF, Wolf SM (2014). Addressing the ethical challenges in genetic testing and sequencing of children. Am J Bioeth.

[68] Friedman JM, Cornel MC, Goldenberg AJ, Lister KJ, Sénécal K, Vears DF (2017). Genomic newborn screening: public health policy considerations and recommendations. BMC Med Genomics.

[69] Geelen E, van Hoyweghen I, Doevendans PA, Marcelis CLM, Horstman K (2011). Constructing “best interests”: genetic testing of children in families with hypertrophic cardiomyopathy. Am J Med Genet A.

[70] Hardart GE, Chung WK (2014). Genetic testing of children for diseases that have onset in adulthood: the limits of family interests. Pediatrics.

[71] Kopelman LM (2007). Using the best interests standard to decide whether to test children for untreatable, late-onset genetic diseases. J Med Philos.

[72] Malpas PJ (2008). Predictive genetic testing of children for adult-onset diseases and psychological harm. J Med Ethics.

[73] Parker M (2010). Genetic testing in children and young people. Fam Cancer.

[74] Ross LF (2013). Predictive genetic testing of children and the role of the best interest standard. J Law Med Ethics.

[75] Ross LF, Clayton EW (2019) Ethical issues in newborn sequencing research: the case study of BabySeq. Pediatrics 144(6). 10.1542/peds.2019-1031 10.1542/peds.2019-1031 PMC6889970 31719124

[76] Flicker LS (2012). Acting in the best interest of a child does not mean choosing the “best” child. Am J Bioeth.

[77] Cherkassky L (2015). The wrong harvest: the law on saviour siblings. International Journal of Law, Policy and the Family.

[78] Cherkassky L (2016). The interfamilial principle and the harvest festival. Eur J Health Law.

[79] Rubeis G, Steger F (2019). Saving whom? The ethical challenges of harvesting tissue from savior siblings. Eur J Haematol.

[80] Birchley G (2014). Deciding together? Best interests and shared decision-making in paediatric intensive care. Health Care Anal.

[81] Catlin A (1996). The dilemma of Jehovah’s Witness children who need blood to survive. HEC Forum.

[82] Redmann AJ, Schopper M, Antommaria AHM, Ragsdale J, de Alarcón A, Rutter MJ, Hart CK, Myer CM (2018). To transfuse or not to transfuse? Jehovah’s Witnesses and postoperative hemorrhage in pediatric otolaryngology. Int J Pediatr Otorhinolaryngol.

[83] Cornfield DN, Kahn JP (2012). Decisions about life-sustaining measures in children: in whose best interests?. Acta Paediatr.

[84] Diekema DS (2004). Parental refusals of medical treatment: the harm principle as threshold for state intervention. Theor Med Bioeth.

[85] Gillam L, Sullivan J (2011). Ethics at the end of life: who should make decisions about treatment limitation for young children with life-threatening or life-limiting conditions?. J Paediatr Child Health.

[86] Jones PM (2016). Thoughtfulness and grace: end-of-life decision making for children with severe developmental disabilities. Am J Bioeth.

[87] Mantulak A (2019). “Best interest” and pediatric end stage kidney disease: the case of baby M. J Pediatr Nurs.

[88] McCradden MD, Anderson JA, Cusimano MD (2019). When is death in a child’s best interest?: examining decisions following severe brain injury. JAMA Pediatr.

[89] Moritz D, Ebbs P (2021). Consent and refusal of treatment by older children in emergency settings. Emerg Med Australas.

[90] Rholl EL, Baughman KR, Leuthner SR (2022). Withdrawing and withholding life-sustaining medical therapies in the neonatal intensive care unit: case-based approaches to clinical controversies. Clin Perinatol.

[91] Savage TA, Michalak DM (2016). When physicians and a parent conflict on when to limit treatment for a child with significant disabilities. Am J Bioeth.

[92] Valdez-Martinez E, Noyes J, Bedolla M (2014). When to stop? Decision-making when children’s cancer treatment is no longer curative: a mixed-method systematic review. BMC Pediatr.

[93] Wellesley H, Jenkins IA (2009). Withholding and withdrawing life-sustaining treatment in children. Paediatr Anaesth.

[94] deSante-Bertkau JE, Haberman B (2017). Resuscitation decisions of extremely premature infants at the limits of viability: defining best interests. Am J Bioeth.

[95] Gillett G (2016). Ashley, two born as one, and the best interests of a child. Camb Q Healthc Ethics.

[96] Taylor M (2018). Too close to the knives: children’s rights, parental authority, and best interests in the context of elective pediatric surgeries. Kennedy Inst Ethics J.

[97] Allmark P, Mason S, Gill AB, Megone C (2001). Is it in a neonate’s best interest to enter a randomised controlled trial?. J Med Ethics.

[98] Di Pietro ML, Cutrera R, Teleman AA, Barbaccia ML (2015). Placebo-controlled trials in pediatrics and the child’s best interest. Ital J Pediatr.

[99] Fofana MO (2013). Joey knows best? Balancing conflicts and defending a child’s best interest in difficult clinical decisions. Virtual Mentor.

[100] Larcher V, Caplan A, Brierley J (2022). COVID-19, children, clinical trials and compassion: the ethical case for using innovative or compassionate treatments. Acta Paediatr.

[101] Litton P (2008). Non-beneficial pediatric research and the best interests standard: a legal and ethical reconciliation. Yale J Health Policy Law Ethics.

[102] Lyons B (2012). Solidarity, children and research. Bioethics.

[103] McGee EM (2003). Altruism, children, and nonbeneficial research. Am J Bioeth.

[104] Morris MC (2012). Pediatric participation in non-therapeutic research. J Law Med Ethics.

[105] Piasecki J, Waligora M, Dranseika V (2015). Non-beneficial pediatric research: individual and social interests. Med Health Care Philos.

[106] Sharav VH (2003). Children in clinical research: a conflict of moral values. Am J Bioeth.

[107] Taylor MJ, Dove ES, Laurie G, Townend D (2018). When can the child speak for herself? The limits of parental consent in data protection law for health research. Med Law Rev.

[108] Bridgeman J (2018). A threshold of significant harm (f)or a viable alternative therapeutic option?. J Med Ethics.

[109] Caplan A, Folkers KM (2017). Charlie Gard and the limits of parental authority. Hastings Cent Rep.

[110] Cave E, Nottingham E (2018). Who knows best (interests)? The case of Charlie Gard. Med Law Rev.

[111] Gillon R (2018). Why Charlie Gard’s parents should have been the decision-makers about their son’s best interests. J Med Ethics.

[112] Schrier L, Hadjipanayis A, Stiris T, Ross-Russell RI, Valiulis A, Turner MA, Zhao W, de Cock P, de Wildt SN, Allegaert K, van den Anker J (2020). Off-label use of medicines in neonates, infants, children, and adolescents: a joint policy statement by the European Academy of Paediatrics and the European Society for Developmental Perinatal and Pediatric Pharmacology. Eur J Pediatr.

[113] Gaucher N, Payot A, Racine E (2013). Cognitive enhancement in children and adolescents: is it in their best interests?. Acta Paediatr.

[114] Wagner K, Maslen H, Oakley J, Savulescu J (2018). Would you be willing to zap your child’s brain? Public perspectives on parental responsibilities and the ethics of enhancing children with transcranial direct current stimulation. AJOB empirical bioethics.

[115] Knoppers BM, Kleiderman E (2019). Heritable genome editing: who speaks for “future” children?. The CRISPR journal.

[116] Cherry MJ (2010). Parental authority and pediatric bioethical decision making. J Med Philos.

[117] Streuli JC, Anderson J, Alef-Defoe S, Bergsträsser E, Jucker J, Meyer S, Chaksad-Weiland S, Vayena E (2021). Combining the best interest standard with shared decision-making in paediatrics-introducing the shared optimum approach based on a qualitative study. Eur J Pediatr.

[118] Anderzén-Carlsson A, Kihlgren M, Svantesson M, Sorlie V (2010). Parental handling of fear in children with cancer; caring in the best interests of the child. J Pediatr Nurs.

[119] Bieber ED, Edelsohn GA, McGee ME, Shekunov J, Romanowicz M, Vande Voort JL, McKean AJS (2020). The role of parental capacity for medical decision-making in medical ethics and the care of psychiatrically ill youth: case report. Front Psych.

[120] Parekh SA (2007) Child consent and the law: an insight and discussion into the law relating to consent and competence. Child Care Health Dev 33(1):78–82. 10.1111/j.1365-2214.2006.00641.x 10.1111/j.1365-2214.2006.00641.x 17181756

[121] Kosher H, Ben-Arieh A (2020). Children’s participation: a new role for children in the field of child maltreatment. Child Abuse Negl.

[122] Kurz R, Gill D, Mjones S (2006). Ethical issues in the daily medical care of children. Eur J Pediatr.

[123] Behrens KG (2020). A principled ethical approach to intersex paediatric surgeries. BMC Med Ethics.

[124] Cannoot P (2021). Do parents really know best? Informed consent to sex assigning and ‘normalising’ treatment of minors with variations of sex characteristics. Cult Health Sex.

[125] Cresti M, Nave E, Lala R (2018). Intersexual births: the epistemology of sex and ethics of sex assignment. Journal of bioethical inquiry.

[126] Kon AA (2015). Ethical issues in decision-making for infants with disorders of sex development. Horm Metab Res.

[127] Newbould M (2016). When parents choose gender: intersex, children, and the law. Med Law Rev.

[128] Wiesemann C, Ude-Koeller S, Sinnecker GHG, Thyen U (2010). Ethical principles and recommendations for the medical management of differences of sex development (DSD)/intersex in children and adolescents. Eur J Pediatr.

[129] Bendorf A, Kerridge IH (2011). Ethical issues in bone marrow transplantation in children. J Paediatr Child Health.

[130] Chan TK, Tipoe GL (2013). The policy statement of the American Academy of Pediatrics—children as hematopoietic stem cell donors—a proposal of modifications for application in the UK. BMC Med Ethics.

[131] Then S-N, Kerridge IH, Marks M (2018). Children as haematopoietic stem cell donors: ethically challenging and legally complex. Med J Aust.

[132] Williamson KA, Vercler CJ (2016). Should children be asked to be bone marrow donors for siblings?. American Medical Association Journal of Ethics.

[133] Bhatia N, Birchley G (2020). Medical tourism and the best interests of the critically ill child in the era of healthcare globalisation. Med Law Rev.

[134] Chervenak FA, McCullough LB, Grünebaum A, Arabin B, Levene MI, Brent RL (2013). Planned home birth: a violation of the best interests of the child standard?. Pediatrics.

[135] Cohen MH, Kemper KJ (2005). Complementary therapies in pediatrics: a legal perspective. Pediatrics.

[136] Gilmour J, Harrison C, Asadi L, Cohen MH, Aung S, Vohra S (2011). Considering complementary and alternative medicine alternatives in cases of life-threatening illness: applying the best-interests test. Pediatrics.

[137] United Nations Committee on the Rights of the Child (2003) General comment No. 5: general measures of implementation of the Convention on the Rights of the Child (CRC/GC/2003/5)

[138] United Nations Committee on the Rights of the Child (2003) General comment No. 4 on adolescent health and development in the context of the Convention on the Rights of the Child (CRC/GC/2003/4)

[139] United Nations Committee on the Rights of the Child (2005) General comment No. 6 on the treatment of unaccompanied and separated children outside their country of origin (CRC/GC/2005/6)

[140] United Nations Committee on the Rights of the Child (2006) General comment No. 9: the rights of children with disabilities (CRC/C/GC/9)

[141] United Nations Committee on the Rights of the Child (2011) General comment No. 13: the right of the child to freedom from all forms of violence (CRC/C/GC/13)

[142] United Nations Committee on the Rights of the Child (2016) General comment No. 19 on public budgeting for the realization of children’s rights (CRC/C/GC/19)

[143] United Nations Committee on the Rights of the Child (2017) General comment No. 21 on children in street situations (CRC/C/GC/21)

[144] United Nations Committee on the Rights of the Child (2021) General comment No. 25 on children’s rights in relation to the digital environment (CRC/C/GC/25)

[145] United Nations Committee on the Rights of the Child and Committee on the Elimination of Discrimination against Women (2019) Joint general recommendation No. 31 and general comment No. 18 on harmful practices (CEDAW/C/GC/31/Rev.1 – CRC/C/GC/18/Rev.1)

